# Evaluating the Implementation of Automated Malaria Rapid Diagnostic Test Readers in Health Facilities in the Democratic Republic of Congo: Process, Challenges, and Lessons Learned

**DOI:** 10.4269/ajtmh.23-0670

**Published:** 2024-10-22

**Authors:** Johanna Karemere, Erick Tshikamba, Erick Mukomena, Curtis Kamwanya, Augustin Guech, Michael Humes, Yazoumé Yé

**Affiliations:** ^1^ U.S. President’s Malaria Initiative Measure Malaria, University of North Carolina at Chapel Hill/ICF, Chapel Hill, North Carolina;; ^2^ U.S. President’s Malaria Initiative, Kinsasha, Democratic Republic of Congo;; ^3^ National Malaria Control Program, Kinsasha, Democratic Republic of Congo;; ^4^ CESMEL Health, Bowie, Maryland;; ^5^ Independent Cybersecurity Consultant, Rockville, Maryland;; ^6^ U.S. President’s Malaria Initiative, Washington, District of Columbia

## Abstract

The WHO’s Global Technical Strategy for malaria emphasizes the importance of reliable malaria surveillance systems to track disease burden and measure progress. A key indicator, the test positivity rate (TPR), largely depends on healthcare providers’ adherence to rapid diagnostic test (RDT) results and their accurate reporting. To minimize healthcare providers’ bias, this study explored the feasibility of using artificial intelligence (AI)–driven Deki Reader devices in the Democratic Republic of Congo. The devices were deployed in 144 health facilities across Haut Katanga, Kasai Central, and Sud Kivu provinces from January to December 2022. Healthcare providers performed malaria diagnostic tests using RDTs and reported the results through the routine system. In addition, they used the Deki Reader device, which automatically read, recorded, and transmitted the AI interpretation into a cloud database. The study compared TPRs from both sources to identify discrepancies. The study revealed the feasibility of using these devices but also identified several logistic and technical challenges. These included delays in device procurement due to COVID-19 pandemic and customs issues, emphasizing the need for better planning and coordination in future rollouts. Device malfunctions and the reliance on stable internet connectivity highlighted the importance of robust support systems and contingency plans. This study demonstrated both the benefits and challenges of implementing such digital health technologies in primary health facilities. Key considerations for successful deployment include careful planning, adequate training and supervision, and taking into account local infrastructure, especially internet connectivity.

## INTRODUCTION

Malaria surveillance is a key component of the WHO’s Global Technical Strategy and relies on robust data collection for informed decision-making. This strategy emphasizes the need for substantial investment in surveillance to reduce the malaria burden.^1^ An effective surveillance system requires enhanced and harmonized case reporting, enabling timely planning, monitoring, and evaluation of progress in malaria control efforts.^2^

Despite significant progress, the malaria community faces challenges in accurately and promptly measuring the malaria burden, particularly in high-transmission countries. The test positivity rate (TPR), a key indicator of malaria transmission risk, depends on healthcare providers accurately reporting malaria diagnostic test results.^3^ However, the literature suggests that low and inconsistent adherence to rapid diagnostic test (RDT) results by health providers is a major barrier in malaria surveillance and control.^4^^,^^5^ Thus, this highlights the need for more reliable and objective methods for interpreting and reporting RDT results, ensuring precise malaria diagnosis, treatment, and burden measurement.

Innovative approaches using new technology, such as the artificial intelligence (AI)–driven Deki Reader devices, are being explored to reduce biases in RDT results interpretation and reporting to improve TPR accuracy.^6^ Studies suggest these automated readers can match healthcare provider’s accuracy in RDT interpretation. Therefore, integrating Deki Reader devices into surveillance systems offers a solution to the challenges of subjective human interpretation and inconsistent adherence to RDT results across settings.^7^ However, there is limited documentation on the feasibility of rolling out such technology in health facilities with different geographic accessibility challenges.^8^^,^^9^

This study assessed the feasibility of rolling out the Deki Reader devices, particularly in high-burden malaria settings with diverse ecological, malaria transmission risk, and geographic accessibility challenges, such as the Democratic Republic of Congo (DRC). Furthermore, previous studies have shown significant discrepancies between TPRs from RDT readers and routine health information system reports in the DRC.^10^ By systematically documenting rollout lessons learned and challenges, this study provides insight into the potential of digital technological solutions in enhancing malaria surveillance and control strategies. This paper covers the implementation process of the RDT study using Deki Reader devices in the DRC from January to December 2022, discussing challenges, lessons learned, and implications for future rollout of similar digital devices. The results of the study estimating the difference between the two reporting methods will be presented elsewhere.

## MATERIALS AND METHODS

### Study sites.

**Figure 1.**
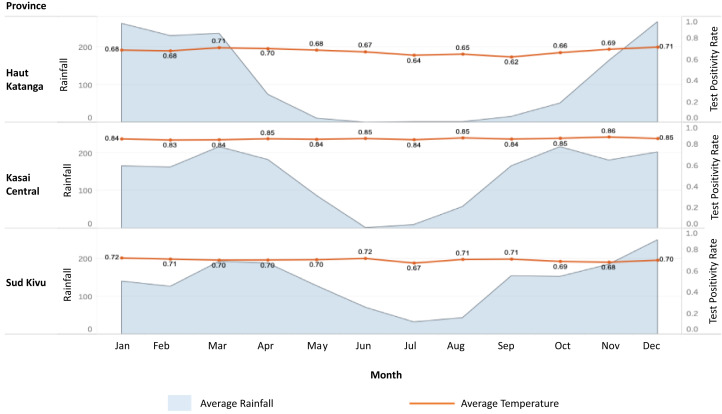
Average monthly precipitation and TPR obtained by RDT for the provinces of Haut Katanga, Kasai Central, and Sud Kivu, 2019. Avg. = average; Rf = rainfall; RDT = rapid diagnostic test, TPR = test positivity rate.

This study was conducted by the U.S. President’s Malaria Initiative Measure Malaria project in three provinces of the DRC, including Haut Katanga, Kasai Central, and Sud Kivu. Covering 144 health facilities across 13 health zones with reliable internet access, the study aimed to understand TPR trends in varying ecological and malaria transmission settings defined by rainfall patterns, average TPR from the DHIS2, and malaria parasite prevalence. These provinces, in the southeastern corridor of the DRC, present varied climates with a low rainy season from May to September and no seasonal variation of RDT TPRs (Figure 1). Haut Katanga has little rainfall during the dry season (May–September), Sud Kivu experiences moderate rain during the same dry season period, and Kasai Central has a pattern in between.

### Study design.

**Figure 2.**
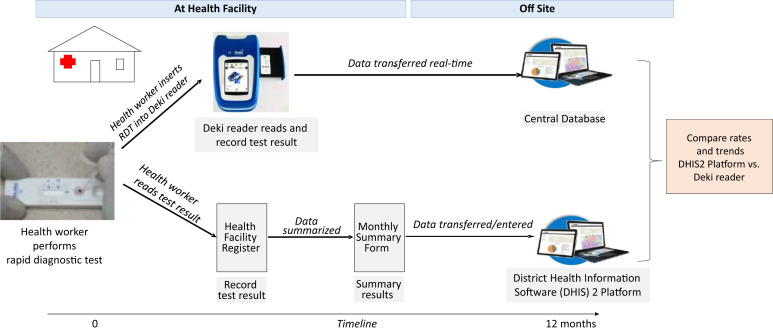
Study design and data flow. RDT = rapid diagnostic test.

This was a prospective study conducted from January through December 2022. Healthcare providers at participating health facilities performed malaria diagnostic tests using RDTs and reported the results through the routine reporting system. In addition, they used the Deki Reader device, which automatically read, recorded, and transmitted the AI interpretation (Figure 2). Importantly, the healthcare providers did not see the Deki Reader device’s AI interpretation of the RDT, and their treatment decisions were based solely on their visual interpretation of the RDT results. After 12 months, the TPRs from both the DHIS2 platform and the Deki Reader device were compared to evaluate any differences.

### Data collection: Data recording using the Deki Readers and reporting into DHIS2.

The Deki Reader is a device that uses AI to interpret RDT results through image analysis and digital data capture software. It features a touchscreen and a user-friendly interface. Each of the 144 selected health facilities in the three provinces was equipped with a Deki Reader device. At these health facilities, healthcare providers performed an RDT on patients and recorded the results in the outpatient department register. These results were later summarized into monthly summary forms and reported on the DHIS2 platform. After making a diagnostic and treatment decision, providers inserted the RDT cassette into the Deki Reader. This device automatically read and processed the RDT result, which was then uploaded together with RDT images, the device’s geo-positioning, and the date and time in real time to a secure online central database via the local mobile phone network. The health facilities in the study primarily used the Bioline^™^ Malaria Ag P.f/P.f/P.v test, compatible with the Deki Reader device.

### Data management and analysis.

Data from the Deki Reader were stored in a central database, accessible to the study team in real time. The study team extracted data monthly from the DHIS2 platform for the 144 health facilities after the health information system team had validated and officially released them, typically in the last week of the following month. Data from the DHIS2 platform were also stored in the same database as the data from the Deki Reader devices. In each province, the study team compared the TPRs from the two sources, accounting for monthly rainfall and mean temperature, which are known to be associated with malaria transmission. The team held meetings to analyze and validate these data to assess the progress of the study.

### Quality assurance strategy.

The study team developed a concise standard operating procedure to rigorously monitor the implementation process, ensuring high-quality outputs. Healthcare providers, responsible for operating the Deki Reader devices, underwent thorough training and received monthly supervision from the study field supervisors. A field supervisor was assigned to each province, responsible for monthly visits to participating health facilities. These visits involved checking the devices’ functionality and supporting healthcare providers with any technical challenges in operating the devices. In addition, the supervisors maintained regular phone contact with providers between visits. After the supervision visit, the study team convened to review data and discuss and resolve any emerging challenges. In addition to visiting health facilities, the Fionet^™^ team and the study’s digital health expert monitored the Deki Reader devices and provided necessary troubleshooting remotely. The digital health expert also ensured data security through restricted access and proper backup measures. Furthermore, the study team reviewed monthly data from participating health facilities reported into the DHIS2 platform to address any data quality issues relevant to the study, such as the completeness of report submissions and key data elements. The study team communicated any identified issues to the health information system team for further action.

### Results of the implementation process coordinating the study.

**Figure 3.**
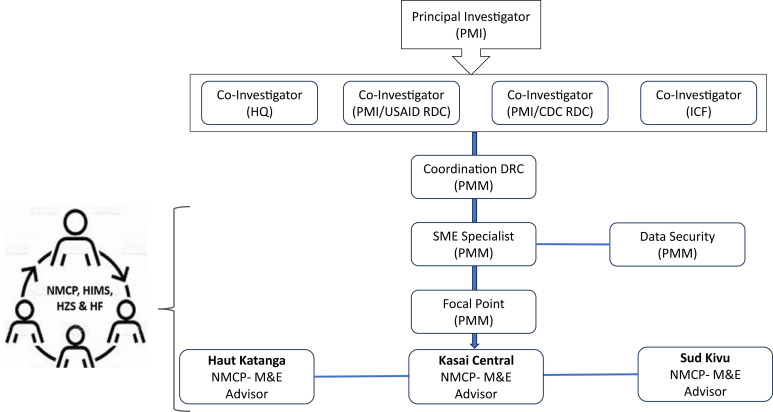
Study team coordination. CDC = Center for Disease Control; DRC = Democratic Republic of Congo; HF = Health Facility; HIMS = Health Information and Management Systems; HQ = Headquarters; HZS = Health Zones; M&E = Monitoring and Evaluation; NMCP = National Malaria Control Program; PMI = President Malaria Initiative, PMM = PMI Measure Malaria Project; SM&E = Surveillance, Monitoring and Evaluation; USAID = United States Agency for International Development.

The principal investigator led the study, working in collaboration with the President Malaria Initiative Measure Malaria Project (PMM) team at headquarters and in the DRC. The in-country study lead (PMM Country Lead) was responsible for implementing the study, working with a field coordinator, three data collection supervisors (who ensured the appropriate use of the device at health facilities) with support from a Fionet consultant, and a digital health expert overseeing the digital technology aspects. Furthermore, the study involved key stakeholders from the Ministry of Health, including the Direction Nationale du Système d’Information Sanitaire and the National Malaria Control Program (NMCP) at the national level, as well as the Bureau de l’Information Sanitaire, Recherche et de la Communication at the provincial level (Figure 3).

### Acquiring and deploying the Deki Readers.

Fionet Corporation (www.fio.com; Toronto, Canada) supplied the Deki Reader devices used in this study. They preconfigured the devices before shipping them to the DRC. The process of getting the devices from the source in Canada to the DRC was challenging and took longer than expected. The study team anticipated receiving 144 devices in the second quarter of 2021; however, owing to production challenges and delays in customs clearance in the DRC, only half of the devices were delivered in late December 2021. This first batch enabled the team to commence the study in two of the three planned provinces, Sud Kivu and Kasai Central. The second batch arrived in February 2022, allowing the team to extend the study to the third province, Haut Katanga.

### Functionality and use of Deki Readers.

The study team members underwent training on the functionality and use of the devices in two phases. The first phase involved training the trainers, targeting managers from the Provincial Health Division (DPS) and project managers, which included provincial advisors and consultants. The second phase covered healthcare providers from participating health facilities. In total, 185 healthcare providers were trained, predominantly certified nurses in charge of health facilities and laboratory technicians. The trained personnel included 45 women and 140 men. Of the 144 devices planned, 120 (83.3%) functioned for more than 10 months. However, there were some manufacturing-related defects (6.9%), broken batteries (15.2%), burnt chargers (12.5%), nonfunctional Deki devices, and frequent internet connectivity issues (Table 1).

**Table 1:** Distribution of the number of Deki Reader devices by functionality issue encountered, 2023

Challenges	Provinces			Number	Percentage (%)
	Haut Katanga	Kasai Central	Sud Kivu		
Total Deki Readers Received	48	48	48	144	–
Deki Readers Replaced Owing to Manufacturing Defects	4	1	5	10	6.9
Deki Readers That Had Batteries Replaced	8	2	12	22	15.2
Deki Readers with Broken Charger and Replaced	6	4	8	18	12.5
Deki Readers That Were Moved Owing to Unstable Internet Connectivity	0	2	10	12	8.3
Deki Readers Not Operational at the End of the Study	10	2	12	24	16.7
Deki Readers That Were Functional throughout the Study Period	38	46	36	120	83.3

### Supervision of health facilities.

The data collection supervisors regularly visited health facilities to ensure effective functioning of the Deki Reader devices, provider proficiency with the RDT technique, and accurate data collection and reporting. The first supervision visit involved visiting all participating health facilities to ensure the devices were in place. For subsequent supervision visits, the team assessed the performance of health facilities based on criteria such as daily use of the Deki Reader device, RDT technique proficiency, adherence to RDT use guidelines, accuracy in recording RDTs in the Deki Reader device, and the number of tests logged compared with the expected monthly average. The team visited each of the 144 health facilities more than four times during the study period.

### Monitoring of the study.

At the health zone level, the management teams actively monitored the health facilities through monthly supervisions and quarterly reviews. At the provincial level, they conducted weekly monitoring by analyzing data reported on the portal, in addition to the monthly joint supervision visits of the DPS and health zone managers to health facilities. At the national level, the study team organized weekly online coordination meetings with NMCP managers from both national and provincial levels. In addition, the study team held biweekly meetings to review progress, discuss challenges, and define action items. Because of the numerous challenges encountered, the study team intensified its monitoring efforts. At the provincial level, the data collection coordinator led 47 meetings with data collection supervisors. These meetings focused on daily use of the Deki Reader device in health facilities, device functionality, and RDT availability. At the central level, the study team held 32 meetings that included the PMM technical team, U.S. President’s Malaria Initiative (PMI) team, and NMCP to discuss ways of further reinforcing the monitoring effort.

### Support from the national level to the provincial level.

In addition to training Deki Reader users, national level staff conducted three coaching visits to the provinces. The first visit, in Haut Katanga, involved NMCP managers, national PMM staff, and a Fionet consultant. They trained providers in four health zones, launched activities, and monitored the initial data collection. The second visit took place in Sud Kivu, where NMCP managers, in conjunction with national PMM staff and a Fionet consultant, trained providers in the Ibanda health zone, troubleshot malfunctioning Deki Reader devices, and resolved most implementation issues. The third visit, by the PMI/DRC team to Haut Katanga province, focused on assessing the level of involvement of the DPS and technical skills of healthcare providers, analyzing the reporting performance of health facilities, identifying bottlenecks, and proposing corrective actions.

### Data and system security.

This study relied on digital devices connected to the cloud; therefore, data security was integrated from the outset of the study. An assessment using NIST SP 800-171A and 800-53A Rev.3 assessed the Fionet system to ensure its compliance with requirements. The digital health consultant proactively identified, managed, and mitigated cyber risks associated with using Deki Reader devices, focusing on three key security domains: users/patients, devices, and data. This initial risk assessment facilitated the establishment of essential security levels for the devices and outlined the potential threat landscape for Deki Readers. Throughout the study period, the team carefully monitored all 144 devices, encompassing user activities, network communications, authentication processes, and access to devices and services. Moreover, the team regularly verified device compliance, including status and configuration, against the study requirements.

## STATISTICAL ANALYSES

The study team actively analyzed data and presented outputs at some of the health facilities. At the provincial level, the study team organized two quarterly reviews in each health zone and three quarterly reviews at the health facility level. These meetings focused on identifying success and challenges and proposing potential solutions to streamline the implementation of the study. This approach significantly enhanced the daily use of devices and decreased the proportion of invalid RDT images. This improvement was primarily due to enhanced capacity of healthcare providers in performing RDTs, timely use of the Deki Reader (immediately inserting the RDT into the device after visually reading the result), consistent supervision, and regular monitoring of RDT images captured on the devices. In addition, at the national level, the study team in collaboration with the NMCP organized a midterm analysis covering data from April to June 2022.

### Study challenges and mitigation actions.

The study encountered several challenges, starting with delays in device procurement due to COVID-19. These delays, along with subsequent delivery and custom clearance issues, were resolved through regular meetings with suppliers and U.S. Agency for International Development’s tax exemption. Device functionality issues, such as breakdowns and unstable internet, were addressed by obtaining replacements from Fionet, changing internet providers, and relocating them to health facilities with better internet connectivity. The issue of healthcare providers not immediately inserting the RDT into the Deki Reader after visual interpretation, but rather waiting until lunchtime, the end of the day, or subsequent days, was addressed through supervision and training. Staff turnover necessitated additional training for healthcare providers. The unstable internet connectivity impacted study monitoring, leading to an increase in physical oversight. Table 2 provides further details on key challenges encountered and mitigation strategies used during the study.

**Table 2:** Summary of the key challenges encountered during the study

Components	Challenges	Mitigation Actions
Procurement of the Devices	The COVID-19 pandemic delayed manufacturing of the device, causing a postponed start from 2020 to December 2021. There were issues with delivery monitoring, customs clearance, and incorrect delivery locations (units sent to Lubumbashi instead of Kinshasa).	Regular meetings with Fionet suppliers were held to track delivery. A tax exemption secured through USAID facilitated customs clearance. Devices intended for Lubumbashi were used there while awaiting those for Kinshasa.
Functioning of the Devices	The devices often broke down and were not fully operational throughout the study period. The supplier replaced the defective devices, but there were delays in supply time; consequently, several health facilities were unable to report data for the entire 12 months.	Fionet provided us with additional reserve devices, enabling us to replace the ones that were broken, particularly the batteries and chargers, along with others that were malfunctioning.
	There were unreliable internet connections in some health zones and health facilities.	Initially, the study team transported the devices to a central location with better internet connectivity to clear hanging images and data. However, because of persistent issues, the team switched internet operators and suppliers as needed. The team also relocated the Deki Reader devices from health facilities with unreliable internet connections to those with better connectivity. Although Sud Kivu’s Miti Murhesa Health Zone was initially selected, internet accessibility issues led to the substitution of five health facilities there with five from the Ibanda Health Zone in the same province.
Health Provider Training	Six of the trained providers were transferred or resigned, specifically in Haut Katanga and Kasai Central, causing a delay or gap in reporting.	To address this issue, the team retrained two more providers from the health facilities, in addition to the health facilities in charge, on the use of the Deki Reader device.
	Batch reading of the RDTs with the Deki Reader was practiced by some healthcare providers who did not immediately insert the RDTs into the device after visual interpretation. Instead, they waited until lunchtime, the end of the day, or subsequent days to do so.	To address this issue, the study team regularly analyzed the data to detect this practice and then conducted supervision and training for the healthcare providers concerned
Study Monitoring	Unreliable internet connections in some of the healthcare facilities impacted the remote monitoring of the Deki Reader devices.	The study team developed a daily data collection template and implemented weekly monitoring. In addition, the team mentored healthcare providers and organized visits to health facilities.
Data Analysis	The quarterly data review by health zone was hindered because of unreliable internet connectivity, leading to delays in data uploading.	The study team extracted data from the portal to facilitate the quarterly data review meetings. health zone management teams’ involvement facilitated implementation, especially for healthcare providers concerned about increased workload.

RDT = rapid diagnostic test; USAID = U.S. Agency for International Development.

## DISCUSSION

This paper presents the roll-out of automated malaria RDT readers in primary health facilities in the DRC. The primary finding of this study was the feasibility of deploying Deki Reader devices across diverse geographic and ecological settings. Although this study provided important insight into healthcare providers’ adherence to RDT results, significant logistic and technical challenges were encountered. The device procurement delays due to the COVID-19 pandemic and customs clearance issues highlighted the need for better coordination and planning in future rollouts. In addition, the occurrence of device malfunctions and the reliance on stable internet connectivity for data transmission underscored the importance of having robust support systems and backup plans. The study also revealed the importance of comprehensive training and regular supervision in ensuring regular, timely, and appropriate use of the Deki Reader devices. The fact that healthcare providers’ treatment decisions were based solely on their interpretation of RDTs, without influence from the Deki Reader’s results, brought an additional challenge to the study because they could not see the value addition to their work. This approach, however, provided a unique opportunity to assess the accuracy of healthcare providers’ RDT interpretation and reporting remotely over a 12-month period, thereby validating the efficacy of the Deki Reader device as a tool for assessing accuracy of malaria surveillance data. It is notable that despite these challenges, the implementation process revealed the adaptability and resilience of the health facilities in incorporating new technology into their workflow. Key to its success was the effective engagement and collaboration of stakeholders, including healthcare providers and the Ministry of Health. This synergy, coupled with NMCP’s ownership, enhanced provider performance and facilitated the prevention of RDT stockouts in participating health facilities. Notably, closer supervision, quarterly data reviews, and reliable internet connectivity emerged as crucial elements in improving technical support and data management. The findings led to several recommendations for any future deployments of the Deki Reader or other AI-driven RDT readers: maintaining consistent communication with healthcare workers, involving all stakeholders in study implementation, ensuring permanent internet connectivity at health centers, establishing a data batch control mechanism, making problem-solving processes more flexible, initiating health facility selection at the grassroots level with a focus on internet connectivity, and simplifying reporting processes using more context appropriate digital technology. For instance, the introduction of a mobile device could potentially alleviate some of the hardware issues experienced with the Deki Reader, particularly in settings where infrastructure challenges are prevalent.

## CONCLUSION

The implications of these findings extend beyond malaria surveillance in the DRC, contributing to the broader discourse on the integration of digital health technologies in resource-limited settings. The study highlights both the potential benefits and the challenges of such integration. Key considerations include the need for robust planning and coordination for the procurement and deployment of the technology. In addition, providing adequate training and supervision is crucial for successful implementation. The study also emphasizes the importance of considering local infrastructure elements, such as internet connectivity, in the deployment of digital health technologies, which should be context/setting appropriate. Therefore, this study serves as an essential reference for policymakers, healthcare practitioners, and researchers engaged in the rollout of digital health solutions in similar contexts.

## References

[1] World Health Organization, 2015. *Global Technical Strategy for Malaria 2016–2030*. Available at: https://www.who.int/malaria/publications/atoz/9789241564991/en/. Accessed October 17, 2019.

[2] Lippeveld T Sauerborn R, 2000. Design and Implementation of Health Information Systems. Geneva, Switzerland: World Health Organization, 15‒32.

[3] Kamau A Mtanje G Mataza C Malla L Bejon P Snow RW. 2020. The relationship between facility-based malaria test positivity rate and community-based parasite prevalence. PLoS One 15: e0240058. 33027313 10.1371/journal.pone.0240058 PMC7540858

[4] Boyce MR O’Meara WP, 2017. Use of malaria RDTs in various health contexts across sub-Saharan Africa: A systematic review. BMC Public Health 17: 470. 28521798 10.1186/s12889-017-4398-1 PMC5437623

[5] Aidoo M Incardona S, 2022. Ten years of universal testing: How the rapid diagnostic test became a game changer for malaria case management and improved disease reporting. Am J Trop Med Hyg 106: 29–32. 34749303 10.4269/ajtmh.21-0643 PMC8733544

[6] Adah P Maduka O Obasi O Doherty O Oguntoye S Seadon K Jalon O Zwingerman N Uhomoibhi P, 2018. The role of the Deki Reader^™^ in malaria diagnosis, treatment and reporting: Findings from an Africare pilot project in Nigeria. Malar J 17: 1–10. 29859093 10.1186/s12936-018-2356-8 PMC5984800

[7] Kalinga AK, , 2018. The use of Fionet technology for external quality control of malaria rapid diagnostic tests and monitoring health workers’ performance in rural military health facilities in Tanzania. PLoS One 13: 12. 10.1371/journal.pone.0208583 PMC6307929 30589853

[8] Soti DO Kinoti SN Omar AH Logedi J Mwendwa TK Hirji Z Ferro S, 2015. Feasibility of an innovative electronic mobile system to assist health workers to collect accurate, complete and timely data in a malaria control programme in a remote setting in Kenya. Malar J 14: 1–8. 26530237 10.1186/s12936-015-0965-z PMC4632488

[9] Oyet C Roh ME Kiwanuka N Orikiriza P Wade M Parikh S Mwanga-Amumpaire J Boum Y 2nd, 2017. Evaluation of the Deki Reader^™^, an automated RDT reader and data management device, in a household survey setting in low malaria endemic southwestern Uganda. Malar J 16: 1–6. 29115991 10.1186/s12936-017-2094-3 PMC5678817

[10] Lechthaler F, , 2019. Trends in reported malaria cases and the effects of malaria control in the Democratic Republic of the Congo. PLoS One 14: 7. 10.1371/journal.pone.0219853 PMC6658057 31344062

